# Auger Recombination and Carrier-Surface Optical Phonon Interaction in Van Der Waals Heterostructures Composed of Graphene and 2D Transition Metal Chalcogenides

**DOI:** 10.3390/ma18030720

**Published:** 2025-02-06

**Authors:** Mounira Mahdouani, Ramzi Bourguiga, Spiros Gardelis

**Affiliations:** 1 Laboratoire de Physique des Matériaux: Structure et Propriétés (LR01ES15), Faculté des Sciences de Bizerte, Université de Carthage, Jarzouna-Bizerte 7021, Tunisia; mannoumah@yahoo.fr (M.M.); ramzi.bourguiga@fsb.ucar.tn (R.B.); 2 Condensed Matter Physics Section, Physics Department, National and Kapodistrian University of Athens, Panepistimiopolis, Zografos, 15784 Athens, Greece

**Keywords:** graphene, transition metal dichalcogenides, surface optical phonon scattering Rate, Van der waals heterostructures, auger scattering rate, WS_2_, WSe_2_, MoS_2_, MoSe_2_

## Abstract

We perform a theoretical investigation of the electron–surface optical phonon (SOP) interaction in Van der Waals heterostructures (vdWHs) formed by monolayer graphene (1LG) and transition metal dichalcogenides (TMDCs), using eigenenergies obtained from the tight-binding Hamiltonian for electrons. Our analysis reveals that the SOP interaction strength strongly depends on the specific TMDC material. TMDC layers generate localized SOP modes near the 1LG/TMDC interface, serving as effective scattering centers for graphene carriers through long-range Fröhlich coupling. This interaction leads to resonant coupling of electronic sub-levels with SOP, resulting in Rabi splitting of the electronon energy levels. We further explore the influence of different TMDCs, such as WS_2_, WSe_2_, MoS_2_, and MoSe_2_, on transport properties such as SOP-limited mobility, resistivity, conductivity, and scattering rates across various temperatures and charge carrier densities. Our analysis confirms that at elevated temperatures and low carrier densities, surface optical phonon scattering becomes a dominant factor in determining resistivity. Additionally, we investigate the Auger recombination process at the 1LG/TMDC interface, showing that both Auger and SOP scattering rates increase significantly at room temperature and higher, ultimately converging to constant values as the temperature rises. In contrast, their impact is minimal at lower temperatures. These results highlight the potential of 1LG/TMDC-based vdWHs for controlling key processes, such as SOP interactions and Auger recombination, paving the way for high-performance nanoelectronic and optoelectronic devices.

## 1. Introduction

Graphene, a two-dimensional material just a few atoms thick, has garnered global interest for its potential in developing next-generation miniaturized and smart electronic devices [1]. However, its lack of a band gap poses challenges, particularly for semiconductor applications [2]. To overcome this limitation, considerable research has focused on methods to induce a band gap in graphene, notably through the creation of Van der Waals (vdW) heterostructures [3]. These heterostructures, composed of graphene and transition metal dichalcogenides (TMDCs), leverage the exceptional electronic characteristics of both materials, offering significant advancements in semiconductor device technology [4,5]. The combination of these two-dimensional (2D) materials has attracted considerable attention due to their potential use in ultrathin, flexible, and transparent electronic and optoelectronic applications [6]. By integrating the optical and photoelectric properties of various materials, vdW heterostructures combine the benefits of direct gap semiconductors with strong electron–phonon coupling and high mobility characteristics typical of semimetals [7,8,9,10]. For instance, when graphene is layered atop a single TMDC layer, it can modify graphene’s intrinsic electronic properties while preserving its Dirac cone structure [11].

Graphene–TMDC heterojunctions, which exhibit strong electron–phonon coupling, are promising for nano-photodetectors [12]. However, chemical doping can negatively impact graphene’s performance, and limitations in photoelectric conversion and the regulation of interlayer interactions pose obstacles for further research on these heterostructures. Interfacial interactions can modify graphene’s electronic properties, as shown by the appearance of satellite Dirac cones when graphene is placed on hexagonal boron nitride substrates. Current research aims to investigate interfacial interactions with various materials to tailor specific electronic properties [13,14,15,16,17,18,19].

Among the studies on 2D/2D Van der Waals heterostructures (vdWHs), the combination of graphene, known for its high carrier mobility, and transition metal dichalcogenides (TMDCs) with semiconducting properties, particularly graphene/MoS_2_ (Gr/MoS_2_), stands out as a promising candidate for various applications. These include electronics [20,21], energy storage [22,23], gas sensors [24,25], and chemical sensors [26,27]. The synergy between graphene’s high mobility and the unique properties of TMDCs enables enhanced performance across these fields.

Two-dimensional (2D) tungsten disulfide (WS_2_) has also gained considerable attention for optoelectronic applications due to its direct bandgap, high carrier mobility, chemical stability, and strong light–matter interactions. The complementary properties of Van der Waals (vdW) heterostructures formed by 2D WS_2_ and graphene, offer promising potential for excitonic optoelectronic performance. However, the strong recombination of excitons in WS_2_ poses a significant challenge in achieving a highly sensitive photodetector [12].

Recently, many graphene-based vdW heterostructures were investigated theoretically and experimentally [28]. However, most studies on graphene heterojunctions have mainly focused on their electronic structures [29], preparation methods [30], and applications [31], with limited research on their electron transport properties and intrinsic mechanisms. To advance the practical use of graphene heterojunctions in nanoelectronic devices, it is crucial to develop new heterojunctions and investigate their electron transport characteristics. Monolayer WS_2_ with its excellent electron mobility and substantial direct bandgap also holds significant potential for various nanodevice applications [12,32].

In Van der Waals heterostructures composed of graphene and 2D transition metal dichalcogenides (TMDCs), electron interactions with SPOs are a key factor influencing electronic properties, such as carrier mobility and scattering rates. When graphene is combined with TMDC layers like MoS_2_ or WSe_2_, the lattice mismatch and the high dielectric environment contribute to enhance electron–SOP coupling. Studies have shown that SOPs, particularly in polar substrates, can strongly be coupled with carriers in adjacent 2D layers, creating a distinct energy dispersion that affects the electronic band structure [13,14,15,16,17,18,19]. This coupling is especially notable in heterostructures on polar substrates such as silicon carbide (SiC) or hexagonal boron nitride (hBN), which facilitate resonant interactions at specific phonon frequencies [13,14,15,16,17,18,19]. By tuning these interactions, researchers aim to optimize device performance in applications ranging from high-speed transistors to optoelectronic components, where carrier dynamics and scattering mechanisms are critical. Recent theoretical and experimental investigations provide insights into how SOP modes can be modulated by the material composition and interlayer distance, offering a pathway to finely control electronic behavior in graphene–TMDC heterostructures [33].

For example, in their study, J. Zhen et al. [34] focused on the high carrier mobility and strong electron–phonon coupling in graphene–WS_2_ heterostructures under hydrostatic pressure. The authors explored how pressure influences charge transfer, Fano resonance, and band structure within these heterostructures using density functional theory (DFT) calculations. The research suggests that graphene–TMD (transition metal dichalcogenide) heterostructures exhibit promising potential for optoelectronic applications due to these unique electronic properties [35].

Theoretical models also provide critical insights, predicting electron relaxation and many-body effects induced by SOP in graphene placed on polar substrates, such as SiC or hBN. These models often use the self-energy framework to explain how energy dissipation occurs due to SOP emission, particularly at high electric fields. Calculations show that in systems like graphene–SiC, the interaction can significantly modify carrier dynamics, including the lifetime and spectral properties of quasiparticles, where electron–SO phonon interactions produce a distinct energy gap, limiting certain energy transitions. This theoretical perspective helps explain experimental findings related to electron transport and thermal dissipation observed in these heterostructures [13,14,15,16,17,18,19].

In combining these findings, both theoretical predictions and experimental validations illustrate how SO phonons can alter the electronic and optical properties of vdW heterostructures. Such interactions are crucial for developing future nanoelectronic and photonic devices that rely on controlled phonon interactions within 2D materials.

Recent studies, both experimental and theoretical, have explored electron–surface optical phonon interactions in Van der Waals (vdW) heterostructures made from graphene and transition metal dichalcogenides (TMDCs), particularly to understand their impact on optoelectronic properties. The interaction between electrons and surface optical phonons within these heterostructures can significantly influence charge and energy transfer, as well as the relaxation dynamics of excitons, which are essential for applications in photodetectors, light-emitting devices, and other nanophotonic systems [12].

In experimental research, charge transfer and exciton–phonon coupling in graphene–TMDC heterostructures were closely analyzed using photoluminescence and Raman spectroscopy. For instance, studies presented by Guillaume et al. have shown that when graphene is combined with a TMDC layer like MoS_2_ or MoSe_2_, interlayer electron transfer occurs rapidly, reducing photoluminescence and modifying exciton lifetime, which is primarily driven by strong exciton–phonon coupling in these vdW interfaces [34].

On the theoretical side, modeling efforts have focused on simulating how phonon polariton quasiparticles formed from coupling between photons and phonons in polar materials behave in vdW structures, often incorporating materials such as hexagonal boron nitride (hBN) that introduce unique hyperbolic phonon polariton modes. These modes offer potential for high optical confinement and controlled light–matter interactions at the nanoscale, which are beneficial for device miniaturization and enhanced energy transfer [35].

Overall, these advances underscore the crucial role of electron–surface optical phonon interactions in tailoring the optoelectronic responses of vdW heterostructures, facilitating their application in next-generation electronic and photonic devices.

Recent advancements in graphene–TMDC heterostructures highlight their potential in high mobility field-effect transistors (FETs), sensors, and modulators. These heterostructures combine graphene’s exceptional conductivity and carrier mobility with the semiconducting and optoelectronic properties of TMDCs, such as MoS_2_ and WSe_2_. Graphene acts as a highly conductive channel, while TMDC layers modulate charge transport through their tunable bandgaps. This synergy was demonstrated to enhance the performance of FETs, achieving mobilities exceeding 8000 cm^2^/Vs, suitable for next-generation high-speed electronics and optoelectronics. Additionally, the heterostructures’ strong light absorption and efficient charge transfer mechanisms enable sensitive photodetection and precise light modulation, making them ideal for optical sensors and modulators [36,37].

In this study, we analyze the interactions between the electronic states of monolayer graphene and the surface optical phonons in a monolayer transition metal dichalcogenide (TMDC) within a graphene/TMDC heterostructure.

Graphene’s electronic states are characterized by its linear energy dispersion near the Dirac points (K and K’), resulting in quasiparticles that behave as massless Dirac fermions. These states exhibit high carrier mobility and chirality, making graphene a model system for exploring quantum and semiclassical transport phenomena. The focus of this model lies in the behavior of these charge carriers as they interact with vibrational modes in the adjacent TMDC layer.

TMDCs, due to their polar nature, host surface optical phonon modes associated with relative atomic displacements within their lattice. These modes generate dynamic electric fields that extend beyond the surface of the TMDC, providing a mechanism for coupling with the electronic states in nearby materials. The frequencies and intensities of these modes depend on the specific TMDC material used (e.g., MoS_2_, MoSe_2_, WS_2_, WSe_2_), as well as on the dielectric environment surrounding the heterostructure.

The interaction at the graphene/TMDC interface is dominated by the long-range Coulomb coupling between the dynamic electric fields from the surface optical phonons and the charge carriers in graphene. Factors such as the proximity of graphene to the TMDC layer, the dielectric mismatch between the materials, and the specific phonon dispersion of the TMDC determine the coupling strength. This interaction modifies the carrier dynamics in graphene, influencing properties like mobility, scattering rates, and energy relaxation.

Understanding these interactions is essential for unraveling the fundamental mechanisms governing interfacial coupling in Van der Waals heterostructures. The insights gained from this model are crucial for optimizing the performance of graphene/TMDC-based devices in applications such as high-frequency transistors, photodetectors, and thermoelectric systems. Additionally, this study sheds light on novel physical phenomena arising from the interplay of Dirac electrons with polar phonons, such as hybrid excitations and non-equilibrium transport.

We investigated theoretically the interaction between phonons and electrons forming polarons within the 1LG/TMDC heterostructures, given that in these material electrons can move without scattering for thousands of interatomic distances [37,38]. The Auger process in graphene-based heterostructures with transition metal dichalcogenides (TMDs) like WS_2_, WSe_2_, MoS_2_, and MoSe_2_ involves the interaction of charge carriers that leads to non-radiative recombination. In these heterostructures, the proximity of graphene to TMDs enhances carrier dynamics, with energy transfer processes such as interlayer charge transfer playing a significant role [39,40].

In graphene/TMDCs systems, the strong interlayer coupling can enable Auger recombination, which involves the transfer of energy from one carrier to another within the TMD layer. This process is influenced by defect states and can be observed through phenomena like photoluminescence quenching under specific temperature conditions [39,40].

These heterostructures provide tunable electronic and optical properties through external factors like electric fields, enabling control over processes such as charge transfer and Auger recombination, which are vital for optimizing device performance in nanoelectronics and photodetectors [39,40].

This paper is organized as follows: First, we investigate the electrical transport in graphene–TMDC heterostructures by estimating SOP-limited mobility, conductivity and resistivity, and the scattering rate in 1LG/TMDC heterostructures. Second, we study theoretically the electron–surface phonon interaction in 1LG/TMDC heterostructures. Finally, we investigate the Auger recombination process in the 1LG/TMDC interface.

## 2. Electrical Transport in 1LG/ TMDC Interface

In this section, we investigate the effects of various TMDCs on the SOP-limited mobility, conductivity and resistivity, and scattering rate in 1LG/TMDC heterostructures. This analysis considers the influence of SOP scattering originating from the TMDCs and examines how these properties change with temperature.

**Figure 1:**
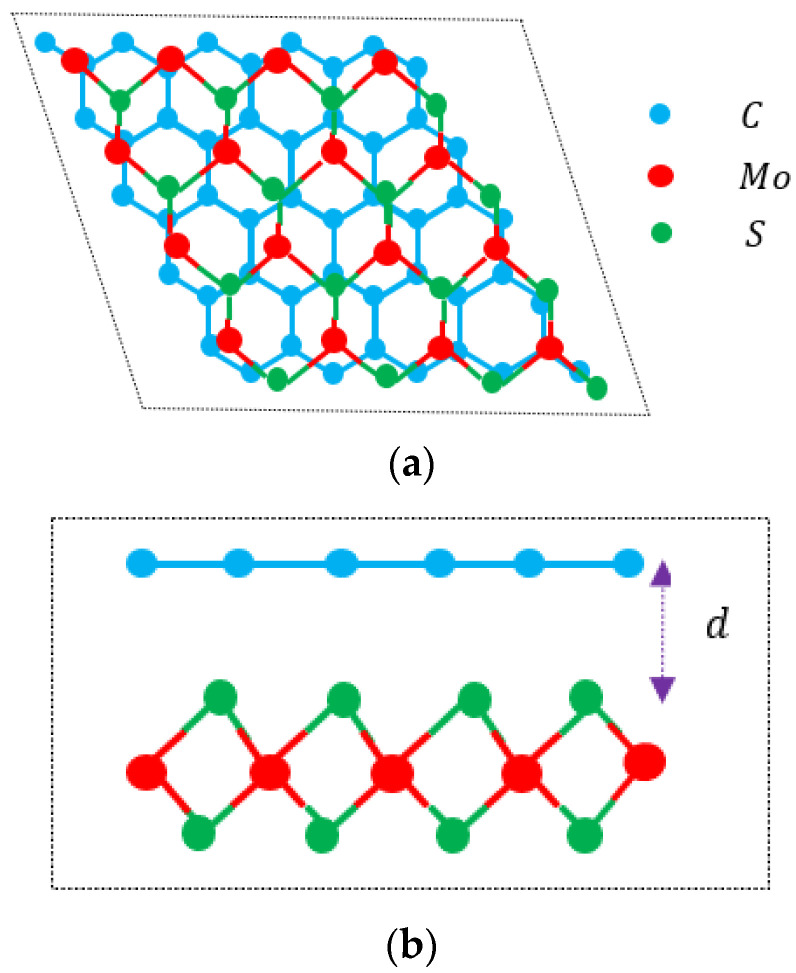
(**a**) Top view of 1LG/MoS_2_ heterostructure. (**b**) Side view of 1LG/MoS_2_ heterostructure. Cyan, green, and red full circles represent C, S, and Mo atoms, respectively. d is vdWHs distance.

In recent years, research on electron mobility within vdWHs made up of graphene and 2D TMDCs (see Figure 1) has expanded significantly. These heterostructures exhibit promising electrical transport properties, influenced largely by the interactions at the graphene–TMDC interface. Electron mobility in these systems is notably high due to graphene’s intrinsic high conductivity, yet it is modified by coupling with the TMDC layer. For example, in graphene/WS_2_ heterostructures, theoretical studies show that electron mobility is enhanced due to the weak interlayer interactions that preserve the Dirac cone structure of graphene while benefiting from the TMDC’s strong spin–orbit coupling properties. Such interactions are pivotal for achieving linear current–voltage characteristics, as noted in heterostructure studies utilizing density functional theory and other advanced simulations [12,33].

Experimentally, electron mobility is also highly dependent on the choice of TMDC material and the heterostructure’s fabrication quality. Variations in stacking angles and interlayer distance can significantly impact heterostructure electron mobility, as they influence the degree of charge transfer and scattering effects at the interface. For instance, research on MoS_2_/graphene heterostructures demonstrates that optimal stacking and minimal interfacial defects allow for a balance between high mobility and controlled charge transfer, thus making these structures suitable for high-performance electronic applications [41].

These findings underscore the potential of graphene–TMDC heterostructures in applications requiring both high electron mobility and tunable electronic properties, such as next-generation photodetectors and flexible electronic devices [12].

Graphene exhibits remarkable electronic properties, particularly its exceptionally high mobility. This characteristic primarily stems from the reduced number of scattering centers in graphene, thanks to the strong covalent chemical bonds that contribute to its extraordinary rigidity. These bonds result in a crystal structure with few defects, that are typically a significant cause of electron scattering. It is important to note that mobility is closely tied to a material’s electrical conductivity and the level of impurities it contains. Higher mobility means that electrons can travel further without colliding with impurities or crystal defects. Additionally, graphene’s high mobility is also intrinsically linked to the unique nature of its electrons, which, unlike those in most materials, behave as massless particles.

The low-field mobility *μ* can be determined by solving the Boltzmann transport equation in the stationary regime, expressed as $\sigma =en\mu =e^{2}v_{F}^{2}D_{n}\tau /2,$ where σ is the electrical conductivity; n is the carrier density; $D_{n}=2E_{F}/(\pi \hbar^{2}v_{F}^{2})$ is the density of states; $E_{F}=\hbar v_{F}\sqrt{\pi n}$ is the Fermi energy; and τ is the scattering time, evaluated using the method described in reference [42].

(1) $$\frac{1}{\tau_{k}}=\frac{2\pi }{\hbar }\sum_{q}{\left|M_{k,k+q}\right|}^{2}\left[1-\cos\left(\theta_{k}-\theta_{k+q}\right)\right]\times \left\{N_{q}\delta \left(E_{k}-E_{k+q}+\hbar \omega_{q}\right)+\left(N_{q}+1\right)\delta \left(E_{k}-E_{k+q}-\hbar \omega_{q}\right)\right\}$$

Here, $N_{q}$ is the Bose–Einstein phonon occupation number, and $\theta_{k}$ is a directional angle of wave vector k. ${\left|M_{k,k+q}\right|}^{2}$ is as follows:

(2) $${\left|M_{k,k+q}\right|}^{2}=\frac{1+ss^{\prime }\cos\left(\theta_{k}-\theta_{k+q}\right)}{2}\frac{4\pi^{2}e^{2}F_{\nu }^{2}}{NAq}e^{-2qd}$$

Here, d represents the Van der Waals distance between 1LG and TMDC.

**Figure 2:**
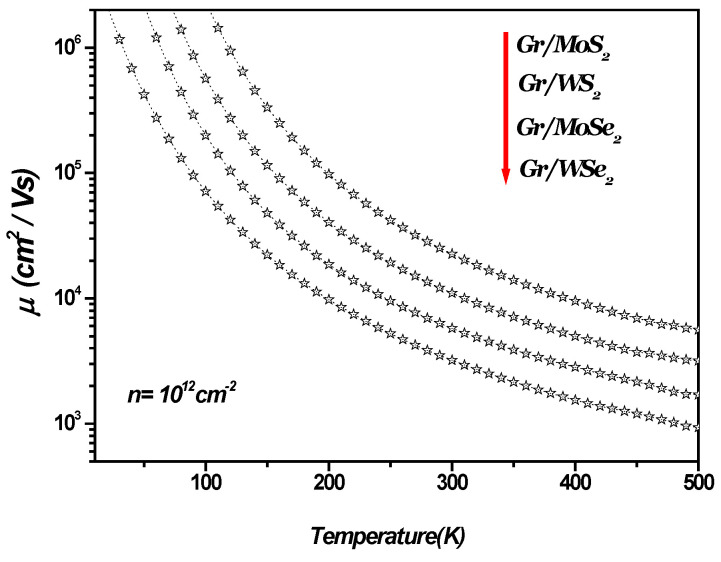
SO phonon-limited mobility versus temperature in 1LG/TMDC (Gr/TMDCs) interfaces. Charge carrier density $n=10^{12}\;{cm}^{-2}$.

Figure 2 illustrates the SO phonon-limited mobility as a function of temperature in monolayer graphene–TMDC heterostructures (1LG/TMDCs). As depicted in Figure 2, SO phonon-limited mobility decreases as the temperature increases depending on the specific type of TMDC.

**Figure 3:**
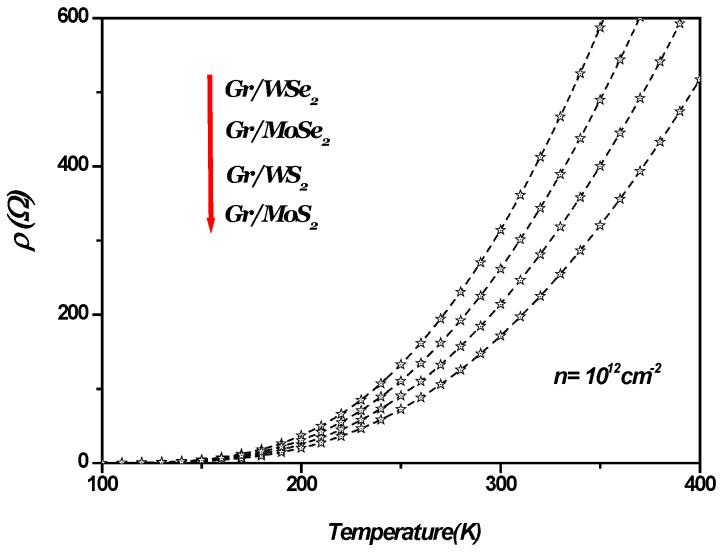
SO phonon-limited resistivity versus temperature in 1LG/TMDC (Gr/TMDCs) interfaces. Charge carrier density $n=10^{12}\;{cm}^{-2}$.

Figure 3 shows SO phonon-limited resistivity versus the temperature in 1LG/TMDC interfaces. As depicted, SOP-limited resistivity increases with increasing temperature. In general, thermal energy causes the vibration of carbon, resulting in an increase in SOP-limited resistivity, thus limiting the maximum conductivity therein. The only way to decrease the resistivity of the material is through significant cooling.

**Figure 4:**
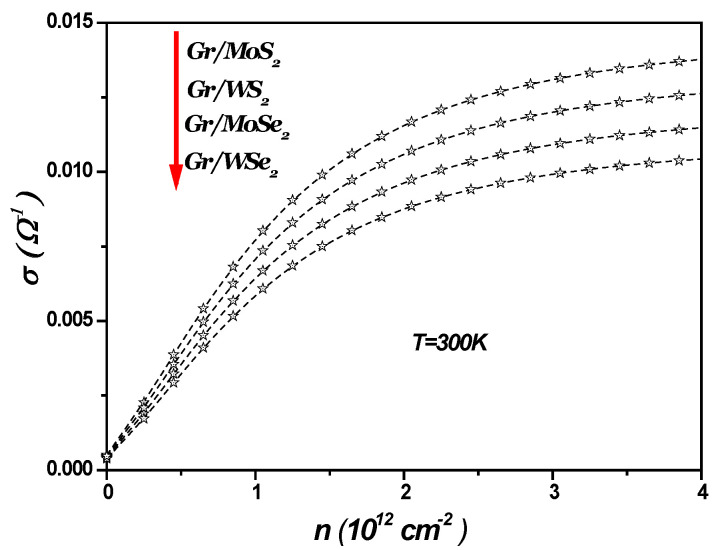
SOP-limited conductivity as function of n in 1LG/TMDC (Gr/TMDCs) interfaces at T = 300 K.

**Figure 5:**
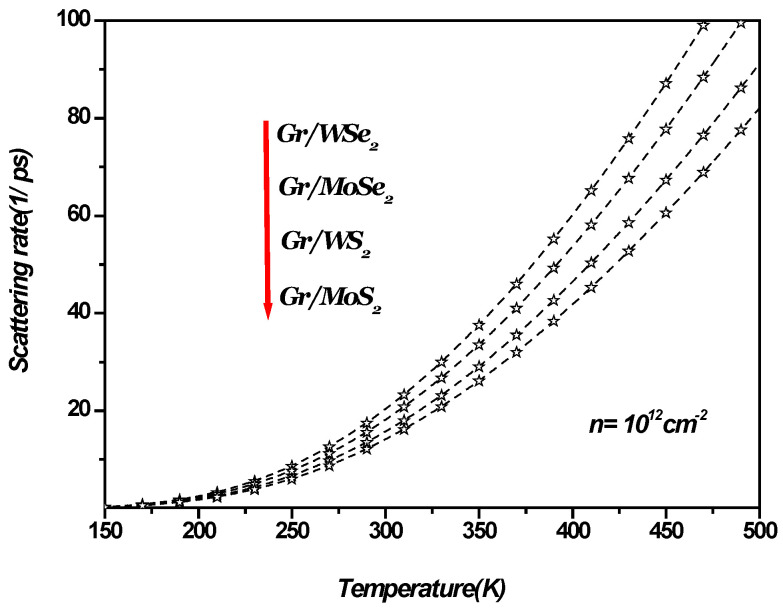
The scattering rate versus the temperature in 1LG/TMDC (Gr/TMDCs) interfaces. The charge carrier density $n=10^{12}\;{cm}^{-2}$.

Figure 4 illustrates SOP-limited conductivity as a function of charge carrier density in a 1LG/TMDC interface at a temperature of 300 K. As the charge carrier density increases, the SOP-limited conductivity becomes more significant, leading to a corresponding decrease in SOP-limited resistivity, given by $\left(\sigma =\rho^{-1}\right)$. Similarly, Figure 5 shows the temperature dependence of the scattering rate in the 1LG/TMDC interface for a charge carrier density of $n=10^{12}\;{cm}^{-2}$. These results indicate that at room and higher temperatures, the SOP scattering rate increases notably, while its impact is minimal at low temperatures. Moreover, at elevated temperatures and low carrier densities, surface optical phonon scattering becomes a dominant factor in determining resistivity [13,14,15,16,42].

The SOP modes at the 1LG/TMDC interface generate an electric field affecting the electrons in the 1LG at distances as far as 4 Å. Remote phonon scattering and its influence on carrier mobility are well known in low-dimensional semiconductor systems and heterostructures [43]. This effect is stronger in graphene due to the significantly smaller vertical dimensions of the devices, governed by the vdW distance.

Transport in one monolayer graphene can be sensitive to the surrounding TMDC. Ideally, TMDCs should have high static dielectric constants and phonon energies that are not activated during low-field transport so that ballistic transport can be achieved in graphene. Thus, TMDCs with high SOP energies and dielectric constants are desirable for applications. For the 1LG/TMDC interface, high temperature transport in graphene is likely dominated by SOP scattering from the TMDCs, as Figure 5 illustrates.

## 3. Electron–Surface Optical Phonon Interaction in 1LG/TMDC Interface

**Figure 6:**
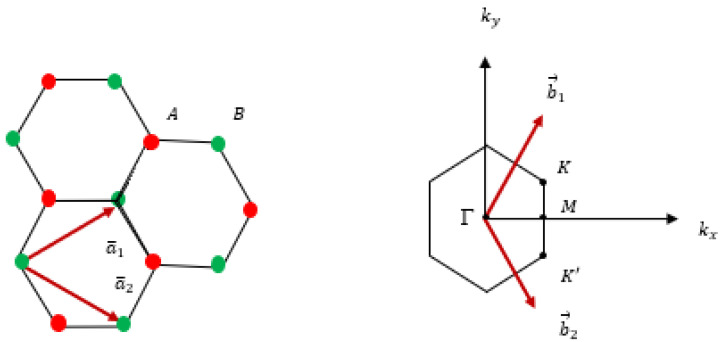
**Left**: The lattice structure of graphene, composed of two interlocking triangular sublattices. The lattice unit vectors are denoted as $a_{1}$ and $a_{2}$. **Right**: The corresponding Brillouin zone, with high-symmetry points marked by black dots. The Dirac cones are situated at *K* and *K*′.

In graphene, the honeycomb lattice does not qualify as a Bravais lattice because the A and B atomic positions are distinct and inequivalent. However, if considered independently, the A (or B) atomic positions form a hexagonal Bravais lattice, often referred to as the “A sublattice” (or “B sublattice”). This configuration can also be described as a triangular lattice with a basis consisting of two atoms per unit cell (refer to Figure 6). The primitive vectors defining the honeycomb lattice are as follows:

$$a_{1}=a_{0}\left(\frac{3}{2},\frac{\sqrt{3}}{2}\right);\;a_{2}=a_{0}\left(\frac{3}{2},-\frac{\sqrt{3}}{2}\right)$$

$a_{0}$ represents the C-C bond distance, approximately 1.42 Å. The reciprocal lattice is defined by the lattice vectors.

$$b_{1}=\left(\frac{2\pi }{3a_{0}},\frac{2\pi }{\sqrt{3}a_{0}}\right);\;b_{2}=\left(\frac{2\pi }{3a_{0}},-\frac{2\pi }{\sqrt{3}a_{0}}\right)$$

*K* and *K*′ at the corners of the graphene Brillouin zone (BZ) are as follows:


$$K=\left(\frac{2\pi }{3a_{0}},\frac{2\pi }{3\sqrt{3}a_{0}}\right);\;K^{\prime }=\left(\frac{2\pi }{3a_{0}},-\frac{2\pi }{3\sqrt{3}a_{0}}\right)$$

In our theoretical study, we employed tight-binding Hamiltonian for electrons at the 1LG/TMDC interface, assuming that electrons can hop to both nearest and next nearest-neighbor atoms. The Hamiltonian has the following form [44]:


(3) $$H=-t_{0}\sum_{R\in A}\sum_{i=1,2,3}c_{R}^{*}c_{R+\delta_{i}}+H.c.$$

Here, “*H*.*c*.” refers to the “Hermitian conjugate”, and $t_{0}\sim 3.1\;eV$ represents the nearest-neighbor hopping energy (the hopping between different sublattices) [44]. The energy bands derived from this Hamiltonian are provided as follows [44]:

(4) $${\;\;\;E\left(k\right)=\varepsilon }_{k\pm \;\;}=\pm \left|t_{k}\right|=\pm t_{0}\sqrt{3+F\left(k\right)}\;$$

where


(5) $$t_{k}=t_{0}\left[1+2exp\left(-i\frac{3k_{x}a_{0}}{2}\right)\cos\left(\frac{\sqrt{3}}{2}k_{y}a_{0}\right)\right]$$

(6) $$F\left(k\right)=4\cos\left(\frac{3}{2}k_{x}a_{0}\right)\cos\left(\frac{\sqrt{3}}{2}k_{y}a_{0}\right)+2\cos\left(\sqrt{3}k_{y}a_{0}\right)$$

In this study, we explore the interaction between electrons and surface optical phonons (SOPs) at the 1LG/TMDC interface, focusing on the long-range Fröhlich coupling. This model provides a robust foundation for comprehending electron–SOP interactions at the 1LG/TMDC interface, but it relies on several assumptions. For instance, it typically applies the Born–Oppenheimer approximation [45]. Short-range interactions, such as electron–phonon interactions in graphene [46], are excluded, and nonlinear interactions and multi-phonon processes are generally neglected [47]. The Fröhlich model also omits the effects of impurities, defects, and other types of disorder that can influence electron–phonon interactions in real materials [45,48]. Additionally, phonon dispersion is usually assumed to be linear, an approximation that may not apply to all phonon modes or substrates [49]. Often, only a single dominant phonon mode is considered, overlooking the potential contributions from multiple phonon modes [46].

For simplicity, we assume an isotropic phonon spectrum, i.e., phonons are either longitudinal or transverse. Fröhlich Hamiltonian includes a term denoting the scattering of an electron from $\vec{k}$ to ${\vec{k}}^{\prime }=\vec{k+q}$, involving the emission or absorption of a phonon. The conservation of the total momentum is maintained, and can be written as follows:


(7) $${H=H}_{ph}+H_{e-ph}$$

The term ${\;H}_{ph}$ represents the phonon energies, which include both the Longitudinal Optical (LO) and SOP modes, and can be written as follows:


(8) $$H_{ph}=\sum_{q,\nu }\hbar \omega_{\nu }{\;a}_{q}^{+}a_{q}$$

In this context, ${\;a}_{q}^{+}$, $a_{q}$ denotes the creation and annihilation operators, respectively, for the phonon with wave vector q, while $\omega_{\nu }$ refers to the frequency of the phonon.

$H_{e-ph}$ is the Hamiltonian describing electron–phonon interaction [50].


(9) $$H_{e-ph}=\sum_{q,\nu }M_{q,\;\;\nu }\left({\;a}_{-q}^{+}{+a}_{q}\right){\;e}^{-iq\;r}$$

Fröhlich Hamiltonian is expressed as follows:


(10) $$H=\sum_{q,\nu }\hbar \omega_{\nu }{\;a}_{q}^{+}a_{q}+\sum_{q,\nu }M_{q,\;\;\nu }\left({\;a}_{-q}^{+}{+a}_{q}\right){\;e}^{-iq\;r}\;$$

The second term in Equation (10) describes the interaction of the electron in monolayer graphene and TMDC-SOP at the interface of the 1LG/TMDCs heterostructure. $M_{q,\;\nu }$ denotes the coupling element in Fröhlich Hamiltonian, describing the interaction between the electron in monolayer graphene and the TMDC’s SOP, and is given by [51,52,53] the following:


(11) $$V_{SOP}{=M}_{q,\;\;SO}=\left|\vec{k}-\vec{k+q}\right|\sqrt{\frac{e^{2}\;F_{\nu }^{2}}{2NAq}}e^{-qz_{0}}$$

In the given context, $F_{\nu }^{2}$ describes the polarization field determined by the Fröhlich coupling [54].


(12) $$F_{\nu }^{2}=\frac{\hbar \omega_{SO,\nu }}{2\pi }\left(\frac{1}{\varepsilon_{\infty }+\varepsilon_{env}}-\frac{1}{\varepsilon_{0}+\varepsilon_{env}}\right)$$

Here, $\varepsilon_{0}$ and $\varepsilon_{\infty }$ are the low- and high-frequency dielectric constants of the TMDCs, (see Table 1), and $z_{0}$ refers to the internal distance between the 1LG and TMDC. The term $\hbar \omega_{SO,\nu }$ denotes the energy of SO phonon of the polar substrates with two branches ν=1, 2.

SOP energies are derived from the bulk LO phonons as follows [44]:


(13) $$\hbar \omega_{SO}=\hbar \omega_{LO}{\left(\frac{1+\frac{1}{\varepsilon_{0}}}{1+\frac{1}{\varepsilon_{\infty }}}\right)}^{\frac{1}{2}}$$

In our analysis, we consider a weak screening of the electric field perpendicular to the TMDC plane and thus the dielectric constant of the environment, $\varepsilon_{env}$, is considered 1 [62].

In the 1LG/TMDC interface, SOPs give rise to an electric field interacting with electrons in the monolayer graphene. From (11) the SOP coupling can be derived and written as follows:


(14) $${W=\sum_{\vec{q}}\left|\left\langle \psi_{k}|V_{SOP}|\psi_{k+q}\right\rangle \right|}^{2}=\frac{NA}{{\left(2\pi \right)}^{2}}\iint \frac{1+ss^{\prime }\cos\left(\theta_{k}-\theta_{k+q}\right)}{2}\frac{4\pi^{2}e^{2}F_{\nu }^{2}}{NAq}e^{-2qz_{0}}qdqd\theta_{q}$$

The summation is performed over one spin and one valley, where $A=\frac{\sqrt{3\;}}{2}a^{2}$ represents the area of the two-atom unit cell.

We have adopted the same method used in our previous computations. [13,14,15,16,17,18,19]. Specifically, to investigate the interactions between electrons and SOPs in monolayer TMDCs, we considered the electronic states. $\left|\psi_{k}^{s}\rangle \right.$ and $\left|\psi_{k+q}^{s\prime }\rangle \right.$, with electron energies $E_{k}=\varepsilon_{k}$ and $E_{k+q}=\varepsilon_{k+q}$, respectively.

The space of polaronic states is obtained from the tensor product of the electronic and phononic state spaces. Therefore, we define new states, referred to as polaronic states, given by the following:


(15) $$\left\{\left|\psi_{k}^{s},1q\rangle \right.\;,\;\;\left|\psi_{k+q}^{s\prime },0q\rangle \right.\right\}$$

The Polaron electron energies $E_{\pm }^{e}$ in the 1LG/TMDC interface are given below [13,14,15,16,17,18,19].

(16) $$E_{\pm }^{e}=\frac{1}{2}\left(E_{k+q}+E_{k}+\hbar \omega_{LO}\right)\pm \sqrt{{\left[\frac{1}{2}\left(E_{k+q}-E_{k}+\hbar \omega_{LO}\right)\right]}^{2}+\frac{NA}{{\left(2\pi \right)}^{2}}\iint \frac{1+ss^{\prime }\cos\left(\theta_{k}-\theta_{q}\right)}{2}\frac{4\pi^{2}e^{2}F_{\nu }^{2}}{NAq}e^{-2qz_{0}}qdqd\theta_{q}}$$

**Figure 7:**
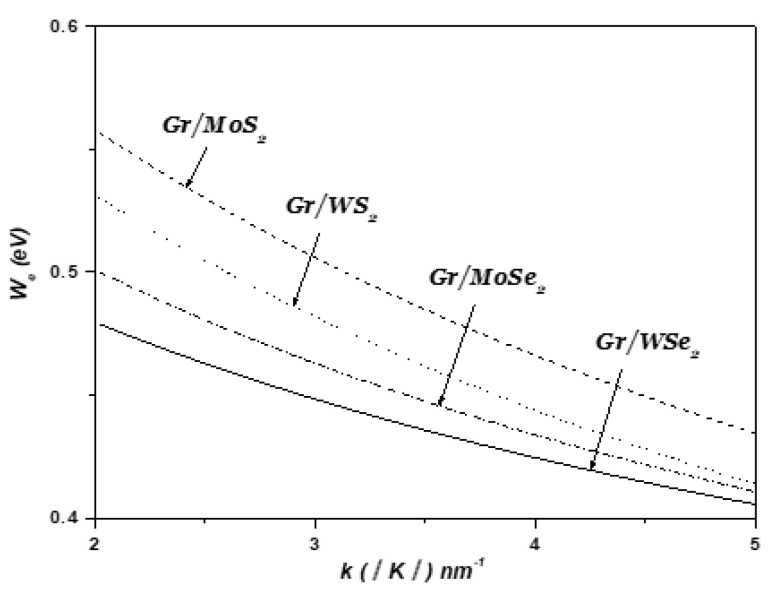
Variation in SOP coupling versus k in 1LG/TMDC interfaces. k changes along Γ-K direction. Kis Dirac point and $\left(\left|K\right|=\frac{4\pi }{{3\sqrt{3}a}_{0}}\right)$.

Figure 7 depicts the strength of the surface optical (SO) coupling between the electronic states $\left|\psi_{k\;}\rangle \right.$ and $\left|\psi_{k+q\;}\rangle \right.$ versus the wave vector k in the 1LG/TMDC interface. As shown in Figure 7, it is clear that the coupling with surface optical phonons (SOPs) is strongly affected by the type of TMDCs.

The observed trends in surface optical phonon (SOP) coupling strength at the graphene/monolayer TMDC interface can be attributed to the intrinsic properties of the TMDCs. The coupling strength is primarily influenced by the SOP frequency, dielectric screening, and polarizability of the TMDC material. S-based TMDCs (e.g., MoS_2_, WS_2_) exhibit higher SOP frequencies and stronger electric fields due to the lighter atomic mass of sulfur, resulting in enhanced coupling with graphene’s Dirac electrons [55,56,57,58,59,60]. In contrast, Se-based TMDCs (e.g., MoSe_2_, WSe_2_) have lower SOP frequencies and greater dielectric screening due to the heavier selenium atoms, leading to weaker coupling [55,56,57,58,59,60,61]. Quantitatively, the coupling strength in S-based TMDCs can exceed that in Se-based TMDCs. These differences highlight the critical role of the TMDC’s lattice dynamics and dielectric properties in determining the strength of electron–phonon interactions at the interface, which in turn influence the carrier mobility, energy dissipation, and overall heterostructure performance.

**Figure 8:**
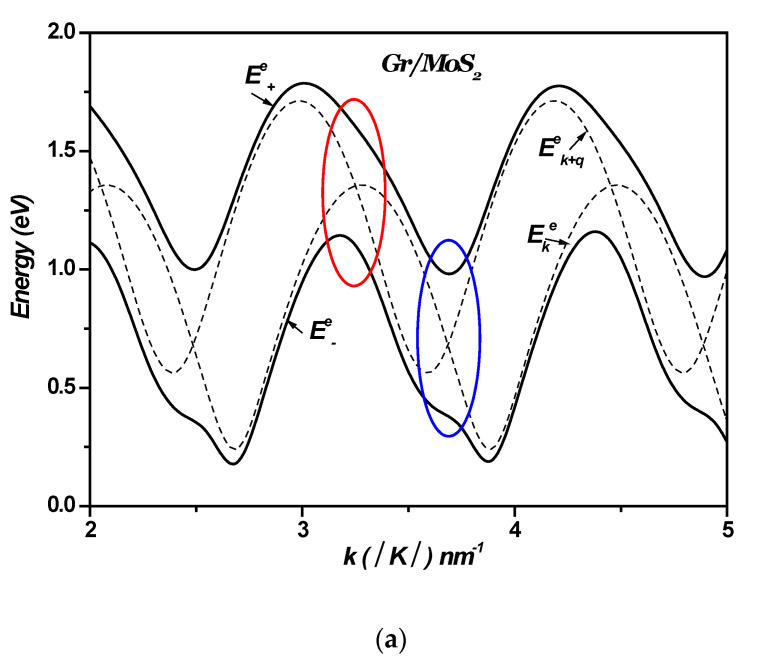

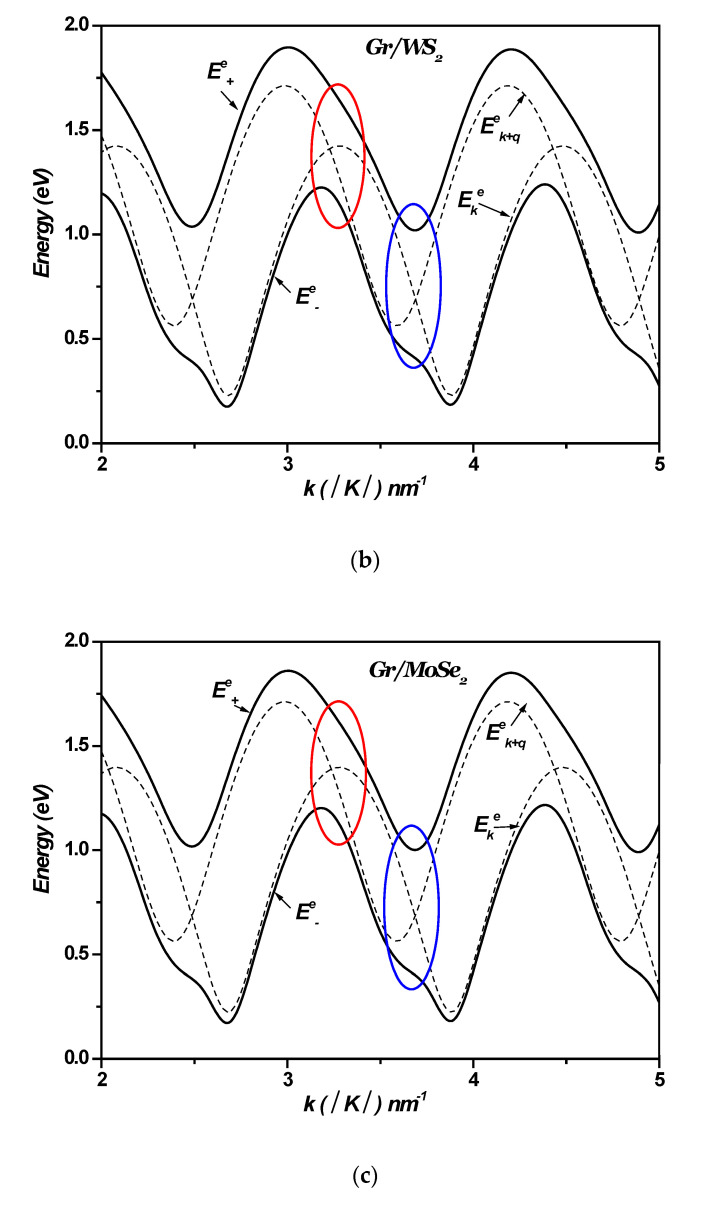

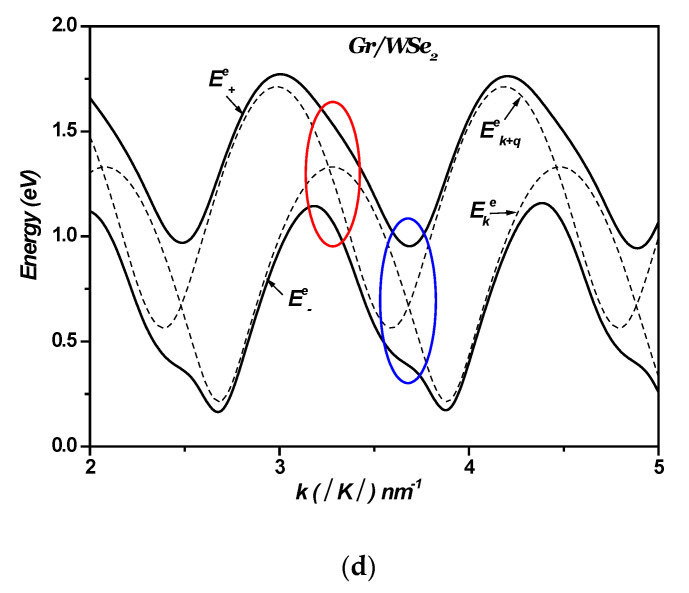
(**a**–**d**). Polaron electron energies vs. *k* in 1LG/TMDC (Gr/TMDCs) interfaces. k changes along Γ-K direction. *K* is Dirac point and $\left(\left|K\right|=\frac{4\pi }{{3\sqrt{3}a}_{0}}\right)$.

**Figure 9:**
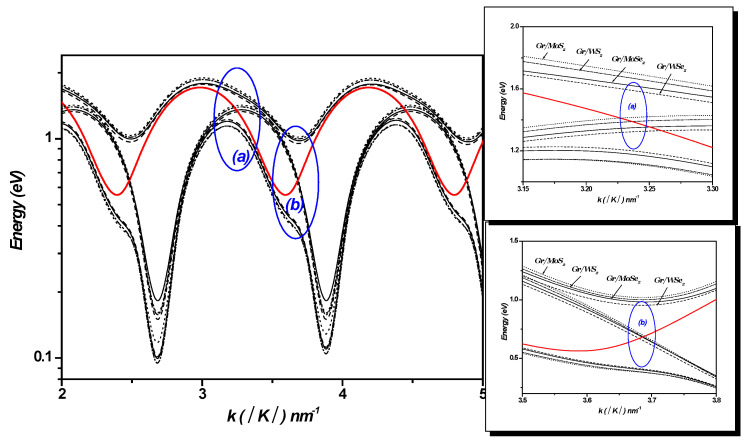
The noninteracting states $\left|\psi_{k}^{s},1q\rangle \right.$ and $\left|\psi_{k+q}^{s\prime },0q\rangle \right.$ cross periodically near $k\sim \left(3.22\pm 1.21\times n\right){\mathrm{nm}}^{-1}$ and $k\sim \left(3.65\pm 1.21\times n\right){\mathrm{nm}}^{-1}$ (n is an integer) in the 1LG/TMDC (Gr/TMDC) interface. k changes along the Γ-K direction. *K* is the Dirac point and $\left(\left|K\right|=\frac{4\pi }{{3\sqrt{3}a}_{0}}\right)$. Right: Zoom-in of the regions (**a**) and (**b**).

Figure 8a–d displays the polaron electron energies as a function of k, with k varying along the Γ-K direction in 1LG/TMDC heterostructures. For comparison, the energies of the noninteracting states are also plotted in the same figures $\left|\psi_{k}^{s},\;1q\rangle \right.$ and $\left|\psi_{k+q}^{s\prime },\;0q\rangle \right.$. These noninteracting levels cross periodically near $k\sim \left(3.22\pm 1.21\times n\right){\mathrm{nm}}^{-1}$ and $k\sim \left(3.65\pm 1.21\times n\right){\mathrm{nm}}^{-1}$ (n is an integer), indicating resonant couplings (see Figure 8 and Figure 9). These crossings imply that the energy separations between the electronic levels are equal to $\hbar \omega_{LO}=46.33\;meV$; $\hbar \omega_{LO}=44.14\;meV$; $\hbar \omega_{LO}=36.95\;meV;$ and $\hbar \omega_{LO}=31\;meV$ for MoS₂, WS₂, MoSe₂, and WSe₂, respectively. They are replaced by large anticrossing energy levels around (~490 meV; ~620 meV), (~460 meV; ~600 meV), (~451 meV; ~590 meV), and (~429 meV; ~569 meV), respectively. The energy values are presented with an associated error margin of ±5 meV. Figure 8 shows that the Rabi splitting of the electron levels becomes larger by changing the TMDCs from MoS_2_ to WS_2_ to MoSe_2_ and finally to WSe_2_ in monolayer graphene–TMDC interfaces.

In these anticrossings, the wave functions of the levels become mixed, enabling multiple transitions such as $E_{k}\to E_{\pm }^{e}\;$, $E_{k}\to E_{k}+\hbar \omega_{LO}$, and $E_{k}\to E_{k+q}$. This indicates that the electron–SOP interaction cannot be regarded as weak coupling. These interactions result in the Rabi splitting of the electron levels. These calculations reveal the likelihood of resonant coupling between the electronic sub-levels and SOPs in the 1LG/TMDC interface.

## 4. Auger Recombination in 1LG/TMDC Interfaces

Auger recombination is a non-radiative mechanism wherein the recombining energy of an electron–hole pair excites another carrier, playing a vital role in reducing photoluminescence efficiency [63,64]. This mechanism affects the performance of light-emitting devices and photodetectors by governing energy transfer dynamics and enabling carrier multiplication, which can enhance device sensitivity under certain conditions. The optical properties arising from Auger recombination in 2D materials, especially TMDCs, are significant for their implications in photonics and optoelectronics. Auger recombination often leads to carrier multiplication or energy dissipation.

To investigate the Auger recombination (AR) process at the 1LG/TMDC interface, we employed the massless Dirac fermion (MDF) Hamiltonian, leveraging the circular symmetry of the system. The semiclassical Boltzmann equation was used, with a collision integral including the effects of electron–electron (e-e) interactions. This approach allowed us to carefully analyze collinear scattering processes, including AR. The behavior of carriers in graphene was described using the MDF Hamiltonian [65,66,67,68].

The behavior of carriers in graphene is governed by the massless Dirac fermion (MDF) Hamiltonian [65,66,67,68].

(17) $${\hat{H}}_{MDF}=\sum_{k,l,s,\sigma }\varepsilon_{k,s}{\hat{\psi }}_{k,l,s,\sigma }^{+}{\hat{\psi }}_{k,l,s,\sigma },$$

Here, the field operator ${\hat{\psi }}_{k,l,s,\sigma }$ annihilates an electron with 2d momentum ħk, valley $l=K,K^{\prime }$, spin σ=↑,↓, band index s=± 1. The quantity $\varepsilon_{k,s}=s\hbar v_{F}\left|k\right|$ represents the MDF band energy, with a slope $\hbar v_{F}≃0.6\;\mathrm{eV}\;\mathrm{nm}.$ MDFs interact through the nonrelativistic Coulomb potential $v\left(r\right)=e^{2}/\left(\bar{\varepsilon }r\right)$ with 2d Fourier transform.

(18) $$v_{q}=\frac{{2\pi e}^{2}}{\bar{\varepsilon }q}$$

Here, $\bar{\varepsilon }=\left(\varepsilon_{1}+\varepsilon_{2}\right)/2$ represents the average dielectric constant [63], determined with the dielectric constants $\varepsilon_{1}$ and $\varepsilon_{2}$ of the media above and below the graphene flake.

Intravalley electron–electron (e-e) interactions are described by the following [69]:


(19) $${\hat{H}}_{e-e}=\frac{1}{2A}\sum_{l}\sum_{\sigma_{1},\sigma_{2}}\sum_{{\left\{s_{i}\right\}}_{i=1}^{4}}\sum_{{\left\{k_{i}\right\}}_{i=1}^{4}}V_{1,2,3,4}^{\left(l\right)}\times \delta \left(k_{1}+k_{2}-k_{3}-k_{4}\right){\hat{\psi }}_{k_{1},l,s_{1},\sigma_{1}}^{+}{\hat{\psi }}_{k_{2},l,s_{2},\sigma_{2}}^{+}{\hat{\psi }}_{k_{4},l,s_{4},\sigma_{2}}{\hat{\psi }}_{k_{3},l,s_{3},\sigma_{1}}$$

Here, *A* denotes the area of the two-dimensional electron system, and the delta distribution implements momentum conservation. The matrix element of the Coulomb potential is expressed as follows:

(20) $$V_{1,2,3,4}^{\left(l\right)}=v_{\left|k_{1}-k_{3}\right|}F_{s_{1},s_{3}}^{\left(l\right)}\left(\theta_{k_{3}}-\theta_{k_{1}}\right)F_{s_{2},s_{4}}^{\left(l\right)}\left(\theta_{k_{4}}-\theta_{k_{2}}\right),$$

where $F_{s_{1},s_{2}}^{\left(l\right)}\left(\theta \right)=\left[1+s_{1}s_{2}exp\left(il\theta \right)\right]/2$ denotes the so-called “chirality factor”, [65,66,67,68], which depends on the polar angle $\theta_{k_{i}}$ of the wave vector $k_{i}$. The strength of electron–electron interactions, relative to the typical kinetic energy, is governed by the following dimensionless coupling constant [67,69]:


(21) $$\alpha_{ee}=\frac{e^{2}}{\hbar v_{F}\bar{\varepsilon }}$$

The Auger scattering rate is expressed as follows [69]:

(22) $$\frac{1}{\tau_{Auger}}=\int_{-\infty }^{+\infty }d\varepsilon_{2}\int_{-\infty }^{+\infty }d\varepsilon_{3}C^{\left(l\right)}\left(\varepsilon_{1},\varepsilon_{3},E\right)\;\left\{\left[1-f_{l}\left(\varepsilon_{1}\right)\right]\left[1-f_{l}\left(\varepsilon_{2}\right)\right]f_{l}\left(\varepsilon_{3}\right)f_{l}\left(\varepsilon_{4}\right)-f_{l}\left(\varepsilon_{1}\right)f_{l}\left(\varepsilon_{2}\right)\left[1-f_{l}\left(\varepsilon_{3}\right)\right]\left[1-f_{l}\left(\varepsilon_{4}\right)\right]\right\}$$

where the Coulomb kernel $C^{\left(l\right)}$, with physical dimensions ${{fs}^{-1}\mathrm{eV}}^{-2}$, stands for the two-particle scattering rate. The energies of the incoming (labeled as 1 and 2) and outgoing particles (labeled as 3, 4) are fixed. The total energy $E\equiv \varepsilon_{1}+\varepsilon_{2}$ is conserved and, finally, $\varepsilon_{4}\equiv E-\varepsilon_{3}$. We note that $f_{l}\left(\varepsilon \right)$ denoted the electron distribution function.

The Auger contribution to the Coulomb kernel, can be expressed as follows [69]:


(23) $${\left.C^{\left(l\right)}\left(\varepsilon_{1},\varepsilon_{3},E\right)\right|}_{Auger}=\frac{1}{8\pi^{2}\hbar^{5}v_{F}^{4}}\sqrt{\frac{\varepsilon_{2}\varepsilon_{3}\varepsilon_{4}}{\varepsilon_{1}}}{\left|V_{1,2,3,4}^{\left(l\right)}\left(k_{1},k_{2},k_{3},k_{4}\right)\right|}^{2}$$

**Figure 10:**
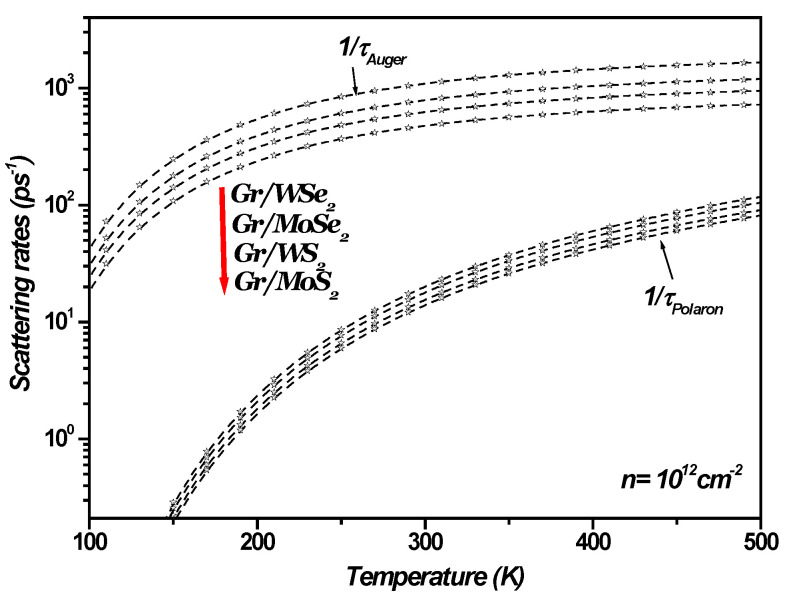
Auger and SOP scattering rates as function of temperature in 1LGr/TMDC interfaces, with charge carrier density of $n=10^{12}\;{cm}^{-2}$.

Figure 10 shows the Auger and SOP scattering rates as a function of temperature in 1LG/TMDC interfaces, with a charge carrier density of $n=10^{12}\;{cm}^{-2}$. The results verify that at room temperature and above, the Auger and SOP scattering rates increase significantly, while at lower temperatures, the effect of both Auger and SOP scattering are minimal.

The calculated scattering rates appear to approach a constant value as temperature (Figure 10) increases. For instance, for the 1LG/MoS_2_ interface, the Auger and SOP scattering rates converge to 789 ps^−1^ and 86 ps^−1^, respectively, corresponding to lifetimes of 1.4 fs and 11.6 fs. This behavior was observed and discussed in references [13,14,15,42], which examine transport in graphene on polar substrates under both low and high bias conditions. Specifically, these studies highlight that low-field mobility converges to a constant value as temperature rises. In the diffusive transport regime, this convergence is attributed to current saturation. While elastic scattering governs low-field mobility, current saturation is linked to inelastic scattering involving either surface polar phonons (SPPs) of the polar substrate or the intrinsic optical phonons of graphene. Furthermore, high bias measurements in graphene, as noted in reference [42], revealed that the magnitude of the saturated current is determined by the energy of the optical phonons responsible for the saturation. Consequently, this saturation induces a convergence of scattering rates in graphene on polar substrates as temperature increases. In the present study, this behavior is demonstrated by the convergence of both Auger and SOP scattering rates at graphene/transition metal dichalcogenide (TMDC) interfaces, as shown in Figure 10.

In graphene/transition metal dichalcogenide (TMDC) heterostructures, current saturation arises primarily due to inelastic scattering mechanisms. Key contributors include interactions with surface optical phonons at the TMDC interfaces and intrinsic optical phonons in graphene. Additionally, electron overheating at elevated electronic temperatures contributes significantly to this saturation. In this state, the system stabilizes in a dynamic equilibrium where further increases in the electric field do not result in higher current.

Beyond transport phenomena, the optical properties of graphene/TMDC interfaces are also significantly influenced by two critical mechanisms: Auger recombination and interactions with surface optical phonons (SOPs). Auger processes, particularly at high carrier densities or in defect-rich materials, can reduce photoluminescence. While this behavior is a limitation for light-emitting devices, it can enhance carrier multiplication in applications such as photodetectors. In hybrid systems like graphene–TMDC heterostructures, interlayer coupling and external fields provide a unique platform to modulate Auger dynamics and phonon interactions, enabling innovative opportunities in quantum and optoelectronic devices.

## 5. Conclusions

In conclusion, firstly, we have investigated the impact of electron–surface optical phonon interactions in monolayer graphene–TMDC heterostructures. For this, we utilized the eigenenergies derived from the tight-binding Hamiltonian. Our study explored the influence of different TMDCs on SOP-limited mobility, conductivity and resistivity, and scattering rates in 1LG/TMDC interfaces, taking into account the effects of SOP scattering. These transport properties are temperature-dependent, with the surface optical phonon scattering becoming more significant at higher temperatures. We have shown that at elevated temperatures, SOP scattering is the dominant scattering mechanism in graphene–TMDC heterostructures. At room temperature and beyond, the SOP scattering rate is notably increased. The surface optical phonon in the graphene–TMDC interface generates an electric field that couples with the electrons in the adjacent graphene. This interaction results in a resonant coupling between the electronic sub-levels and the SOPs, causing the Rabi splitting of electron levels. In summary, our findings indicate that the electron–surface optical phonon interaction is significantly influenced by the choice of TMDC.

Secondly, we have theoretically demonstrated that at room temperature and above, both Auger and SOP scattering rates at the 1LG/TMDC interfaces increase significantly, eventually converging to constant values as the temperature rises. In contrast, at lower temperatures, the impact of both Auger and SOP scattering is minimal. In conclusion, our findings emphasize that Auger recombination and SOP interactions are strongly influenced by the choice of specific TMDC material.

Finally, Van der Waals heterostructures (vdWHs) combining monolayer graphene (1LG) with transition metal dichalcogenides (TMDCs) exhibit outstanding electronic and optical properties, making them promising candidates for next-generation nanoelectronic and optoelectronic devices. The performance of these heterostructures is significantly influenced by electron–surface optical phonon (SOP) interactions and Auger recombination processes, which govern charge carrier dynamics.

## Figures and Tables

**Table 1:** Parameters for surface optical phonons scattering of TMDCs.

	${MoS_{2}}^{a,b}$	${WS_{2}}^{a,b}$	${Mo{Se}_{2}}^{a,b}$	${WSe_{2}}^{a,c}$
${\hbar \omega }_{LO}\;\left(\mathrm{meV}\right)$ $\varepsilon_{0}$ $\varepsilon_{\infty }$ d(Å)	46.33 9.8 9.69 3.38	44.14 9.34 9.24 3.40	36.95 11.19 10.99 3.50	3.1 10.74 10.64 3.87

Referencesa [55,56,57,58,59].  Referenceb [60].  Referencec [61].

## Data Availability

The data presented in this study are available on request from the corresponding author. The data are not publicly available due to privacy issues.
